# Cutaneous Larva Migrans Presenting as Eczematous Dermatitis

**DOI:** 10.1002/ccr3.72983

**Published:** 2026-06-17

**Authors:** Mahesh Mathur, Sumit Paudel, Shilpa Maharjan, Himanshu Pathak, Sandhya Regmi, Sharad Shrestha

**Affiliations:** ^1^ Department of Dermatology College of Medical Sciences Bharatpur Nepal

**Keywords:** creeping eruption, cutaneous larva migrans, eczematous dermatitis, serpiginous rash

## Abstract

Cutaneous larva migrans (CLM) is a skin infection caused by animal hookworm larvae. Clinically, it appears as a red, itchy, winding (serpiginous) line, but early lesions can mimic eczema. We report a case of a 66‐year‐old farmer who initially presented with an eczematous lesion on the left foot, which was subsequently diagnosed as CLM following the appearance of a characteristic serpiginous track. The lesions resolved with albendazole treatment. This case highlights the need to consider CLM in persistent, itchy rashes, especially with occupational exposure to contaminated soil.

## Key Clinical Message

Cutaneous larva migrans can initially present as eczematous dermatitis before the characteristic serpiginous track appears. A high index of suspicion, especially with relevant environmental exposure, enables timely diagnosis and prompt treatment with appropriate therapy, resulting in rapid resolution and prevention of prolonged morbidity.

**Abbreviation**
CLM: Cutaneous Larva Migrans

## 1 Introduction

Cutaneous larva migrans (CLM) is caused by the larvae of hookworms that infect dogs and cats, most commonly *Ancylostoma braziliense* and *Ancylostoma caninum*, and less frequently by *Necator americanus* , *Uncinaria stenocephala*, and *Strongyloides stercoralis* [1]. These larvae are typically found in warm, moist soil or sand contaminated with animal feces and are prevalent in tropical and subtropical climates. Human infection occurs when larvae penetrate intact skin, most commonly involving the feet or lower extremities.

Once inside the epidermis, the larvae migrate, forming winding, erythematous, intensely pruritic tracks known as “creeping eruption” [2]. The incubation period ranges from a few days to several weeks. Early lesions may present as pruritic papules or plaques resembling eczema or insect bites, leading to misdiagnosis in up to 58% of cases [3]. Topical corticosteroids are frequently prescribed but do not eliminate the larvae and may delay appropriate treatment [4].

## 2 Case History/Examination

A 66‐year‐old man presented with a 10‐day history of intensely pruritic, erythematous, scaly papules on the dorsum of his left foot. He was initially diagnosed with eczematous dermatitis and treated with topical corticosteroids and oral antihistamines from Day 3 of symptoms. Despite treatment, symptoms persisted. On Day 10 of clinical course, the patient noticed a red, wavy linear lesion extending from the dorsum of the left foot toward the medial aspect of the ankle. He reported frequent barefoot exposure during agricultural work.

**FIGURE 1:**
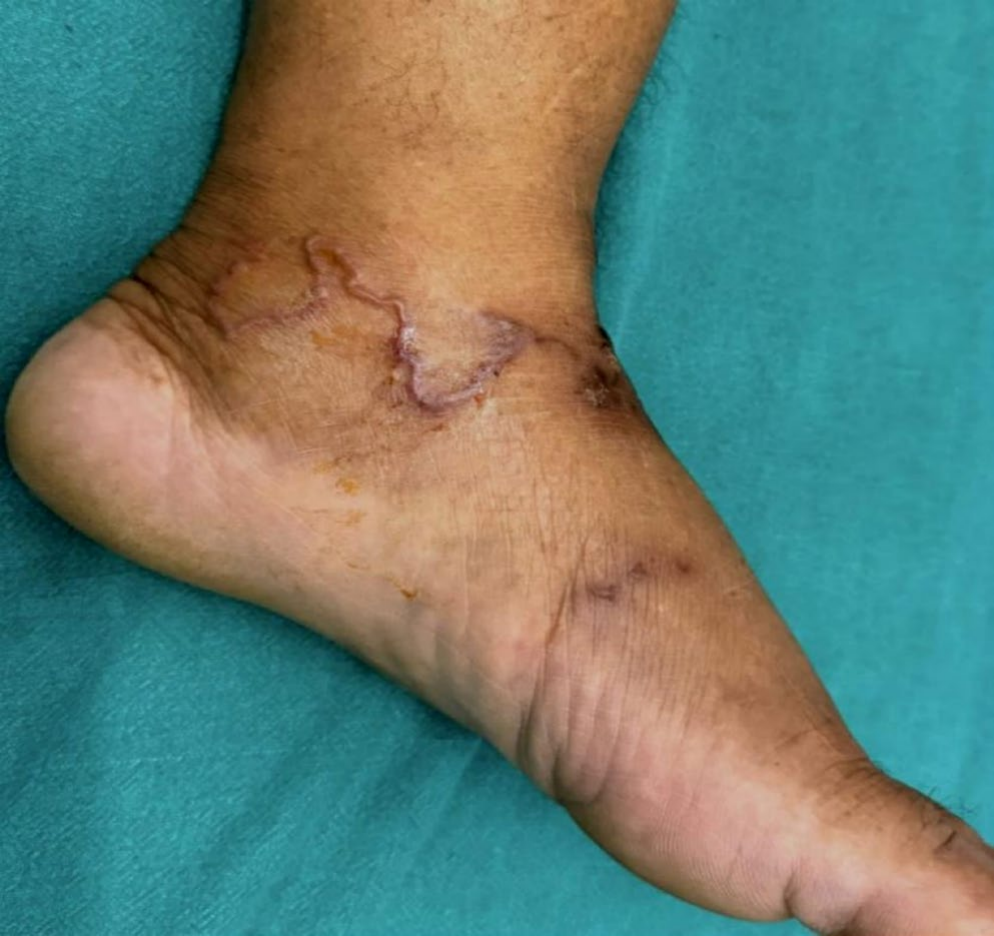
Cutaneous larva migrans (creeping eruption): Well‐defined, raised, serpiginous, erythematous‐to‐violaceous track over the medial aspect of the left ankle on Day 10 of presentation, measuring approximately 6 cm in length.

On examination, a well‐defined, raised, serpiginous erythematous track with excoriations was noted over the medial aspect of the left ankle (Figure 1). No systemic symptoms were present.

## 3 Differential Diagnosis, Investigations, and Treatment

The initial differential diagnoses included eczematous dermatitis, insect bite reaction, allergic contact dermatitis, and cutaneous larva migrans. Based on the characteristic serpiginous lesion and history of barefoot exposure, a clinical diagnosis of cutaneous larva migrans was made.

No laboratory investigations or skin biopsy were performed.

The patient was treated with albendazole 400 mg orally once daily for 5 days, along with oral antihistamines. Albendazole was selected due to its established efficacy, favorable safety profile, and availability in the local setting. Alternative effective regimens include single‐dose ivermectin (200 μg/kg orally) and topical thiabendazole [4, 5].

## 4 Conclusion and Results (Outcome and Follow‐Up)

By the fifth day of treatment, the lesion had flattened with marked reduction in pruritus. At two‐week follow‐up, complete resolution was noted, with only post‐inflammatory hyperpigmentation remaining.

This case highlights how CLM may initially present like eczema or insect bites, leading to misdiagnosis. Recognizing the characteristic serpiginous (winding) rash and obtaining a relevant occupational history are crucial for accurate diagnosis. Prompt treatment with albendazole results in rapid resolution and prevents complications.

## 5 Discussion

CLM is commonly seen in tropical and subtropical regions of Africa, South America, Southeast Asia, the Caribbean, and the southeastern United States, with higher prevalence during the wet season [1]. However, it can occur anywhere contaminated soil is present. The larvae migrate within the epidermis at a rate of approximately 2 cm per day, triggering a strong immune response and causing intense pruritus [2].

Early signs of infection often resemble eczema, insect bites, or allergic dermatitis, leading to diagnostic confusion [3]. Scratching may result in excoriations and secondary bacterial infection [4]. Diagnosis is primarily clinical, based on a history of environmental exposure and the presence of a characteristic serpiginous track. Laboratory tests and skin biopsies are rarely required for typical cases.

Albendazole 400–800 mg daily for 1–7 days is the preferred treatment for CLM. Oral ivermectin and topical thiabendazole are also effective alternatives [5]. Prevention includes wearing shoes and avoiding direct contact with contaminated soil or sand.

## Author Contributions

**Mahesh Mathur:** conceptualization, project administration, supervision, validation. **Sumit Paudel:** conceptualization, data curation, investigation, resources, supervision, writing – original draft. **Shilpa Maharjan:** formal analysis, methodology, visualization, writing – review and editing. **Himanshu Pathak:** funding acquisition, investigation, software, visualization. **Sandhya Regmi:** conceptualization, project administration, supervision. **Sharad Shrestha:** conceptualization, data curation, formal analysis, methodology, supervision, writing – original draft, writing – review and editing.

## Funding

The authors have nothing to report.

## Ethics Statement

Reviewed and approved by Institutional review board College of medical sciences (IRBCOMS). The patients in this manuscript have given written informed consent to the publication of their case details.

## Consent

The authors obtained written consent from the patient for use of photographs and medical information to be published online and with the understanding that this information may be publicly available and discoverable via search engines. Patient consent forms are not provided to the journal but are retained by the authors.

## Conflicts of Interest

The authors declare no conflicts of interest.

## Data Availability

The data that support the findings of this study are available from the corresponding author upon reasonable request.
